# Use of mHealth to Increase Physical Activity Among Breast Cancer Survivors With Fatigue: Qualitative Exploration

**DOI:** 10.2196/23927

**Published:** 2021-03-22

**Authors:** Elise Martin, Antonio Di Meglio, Cecile Charles, Arlindo Ferreira, Arnauld Gbenou, Marine Blond, Benoit Fagnou, Johanna Arvis, Barbara Pistilli, Mahasti Saghatchian, Ines Vaz Luis

**Affiliations:** 1 Institute Gustave Roussy Villejuif France; 2 Kiplin Nantes France

**Keywords:** mHealth, physical activity, breast cancer, cancer-related fatigue, qualitative study, survivorship

## Abstract

**Background:**

Physical activity has shown beneficial effects in the treatment of breast cancer fatigue; nevertheless, a significant portion of patients remain insufficiently physically active after breast cancer. Currently most patients have a smartphone, and therefore mobile health (mHealth) holds the promise of promoting health behavior uptake for many of them.

**Objective:**

In this study, we explored representations, levers, and barriers to physical activity and mHealth interventions among inactive breast cancer patients with fatigue.

**Methods:**

This was an exploratory, qualitative study including breast cancer patients from a French cancer center. A total of 4 focus groups were conducted with 9 patients; 2 independent groups of patients (groups A and B) were interviewed at 2 consecutive times (sessions 1 to 4), before and after their participation in a 2-week mHealth group experience consisting of (1) a competitive virtual exercise group activity (a fictitious world tour), (2) participation in a daily chat network, and (3) access to physical activity information and world tour classification feedback. We used a thematic content analysis.

**Results:**

Several physical activity levers emerged including (1) physical factors such as perception of physical benefit and previous practice, (2) psychological factors such as motivation increased by provider recommendations, (3) social factors such as group practice, and (4) organizational factors including preplanning physical activity sessions. The main barriers to physical activity identified included late effects of cancer treatment, lack of motivation, and lack of time. The lack of familiarity with connected devices was perceived as the main barrier to the use of mHealth as a means to promote physical activity. The tested mHealth group challenge was associated with several positive representations including well-being and good habit promotion and being a motivational catalyzer. Following feedback, modifications were implemented into the mHealth challenge.

**Conclusions:**

mHealth-based, easily accessed group challenges were perceived as levers for the practice of physical activity in this population. mHealth-based group challenges should be explored as options to promote physical activity in a population with fatigue after breast cancer.

## Introduction

There are over 2 million new cases of breast cancer diagnosed worldwide each year, and 80% to 90% of the patients will be alive and free of disease 5 years after diagnosis [1]. In this setting, a focus on management of late and long-term physical, cognitive, psychological, and social effects of cancer and cancer treatment has emerged in the last decade [1-5]. Cancer-related fatigue is reported in up to 50% of breast cancer patients after treatment [1-4,6] and negatively impacts overall quality of life (QoL) of breast cancer patients [6,7].

Several interventions have proven to be effective in reducing cancer-related fatigue among breast cancer survivors and are recommended by cancer societies including the National Comprehensive Cancer Network, Oncology Nursing Society, and American Society of Clinical Oncology [8-10]. Among strategies to decrease cancer-related fatigue, physical activity has been supported by several studies [11-18]. A meta-analysis of 27 exercise intervention studies showed that exercise led to a reduction of cancer-related fatigue with a mean effect size of 0.32 (95% CI 0.21-0.43) during cancer treatment and 0.38 (95% CI 0.21-0.54) following treatment completion. Therefore, it is now recommended that patients, including those experiencing cancer-related fatigue, get at least 150 minutes of moderate intensity aerobic physical activity or 75 minutes of vigorous intensity aerobic physical activity per week or an equivalent combination [19,20]. It is also well documented that fatigue can be a barrier to physical activity engagement [21]. Nevertheless, research suggests that cancer-related fatigue is largely underreported and undertreated [11], and a substantial proportion of breast cancer survivors are inactive during and after treatment [22,23].

Currently a vast proportion of breast cancer patients have smartphones and can easily access the internet [24]. Mobile health (mHealth) uses mobile technology to deliver and share personalized health information and holds the promise of becoming a way to deliver behavioral interventions that are embedded into individuals’ daily routines, with the great potential to reach diverse populations and of being generalizable [25-28]. Some feasibility studies using mHealth to empower breast cancer patients and survivors have been conducted, and some presented promising results [29-35]. Uhm et al [36] conducted a prospective multicenter trial examining the effect of an mHealth-based exercise intervention among breast cancer patients that suggested this strategy could be effective in increasing physical activity in this population. Several companies are designing mHealth options to monitor patient-reported outcomes and promote engagement in health behaviors such as physical activity. Recently, Kiplin, a company in France, developed an mHealth group challenge that provides patients the opportunity of engaging in virtual exercise group challenges [37].

We conducted a qualitative study to explore representations, levers, and barriers to physical activity and mHealth interventions among patients with breast cancer and cancer-related fatigue. Our overarching goal was to explore mHealth as a facilitator to increase physical activity in patients with fatigue after breast cancer. In addition, we tested satisfaction with the Kiplin mHealth group challenge among this population.

## Methods

This qualitative study was conducted following the Consolidated Criteria for Reporting Qualitative Studies (COREQ) [38].

### Participants

Eligible participants had a diagnosis of stage I to III breast cancer according to the American Joint Committee on Cancer version 8 and were followed at a French comprehensive cancer center. Patients were invited to participate by the treating physician if they reported (1) cancer-related fatigue rated as equal or higher than 4/10 on a visual analog scale, (2) declared they did not meet the World Health Organization recommendations for physical activity (ie, 150 or 75 minutes per week of moderate or vigorous activity or equivalent combinations) [19], (3) had a smartphone with internet access, (4) spoke French fluently, and (5) had no physical or medical contraindications to the proposed activity. All patients should have completed breast cancer primary treatment between 3 and 18 months before the first group meeting. We used purposive sampling. Health care professionals asked patients if they were willing to participate in the study when they were at the outpatient clinic. Patients interested in participating were contacted by a trained PhD sociologist (EM) by email or phone call, who would introduce herself and explain the study. In addition, all patients received written information explaining the objectives and the process of the focus group.

### Procedures and Data Collection

A total of 4 focus groups were conducted by EM (sociologist, PhD, experienced in qualitative study) assisted by ADM (medical oncologist, MD, experienced in survivorship and cancer care) between June and November 2018 at the cancer center and lasted on average 90 minutes. Two independent groups of patients were interviewed 2 consecutive times (group A session 1 and 2 and group B, session 3 and 4).

A focus group guide was developed for each interview with diverse stakeholder input, including medical oncologists, psychologists, researchers, and breast cancer survivors who reviewed the content and topic areas and provided feedback (Multimedia Appendices 1 and 2).

### Levers and Barriers to Physical Activity and mHealth Use

The first focus sessions of each group (sessions 1 and 3) were designed to explore physical activity and mHealth use representations, levers, and barriers. In the end of the focus group, instructions for an mHealth group challenge were given, followed by 2 weeks of participation in the actual challenge.

### Kiplin mHealth Group Challenge

The second focus groups (sessions 2 and 4) were performed within 2 weeks of the end of the mHealth-based physical activity challenge and designed to evaluate satisfaction with the mHealth group challenge. As prespecified in the study protocol, the first patient group (group A) feedback led to changes to the challenge for the second one (group B). All participants completed a brief survey that assessed sociodemographic and clinical information on the day of the first focus group. Details of this survey are provided in Multimedia Appendix 3.

### Intervention

**Figure 1:**
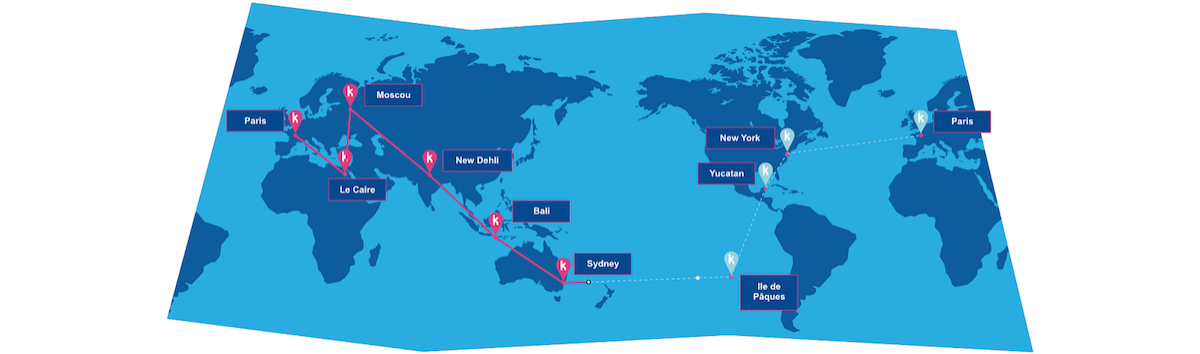
Map (Kiplin’s world tour).

**Figure 2:**
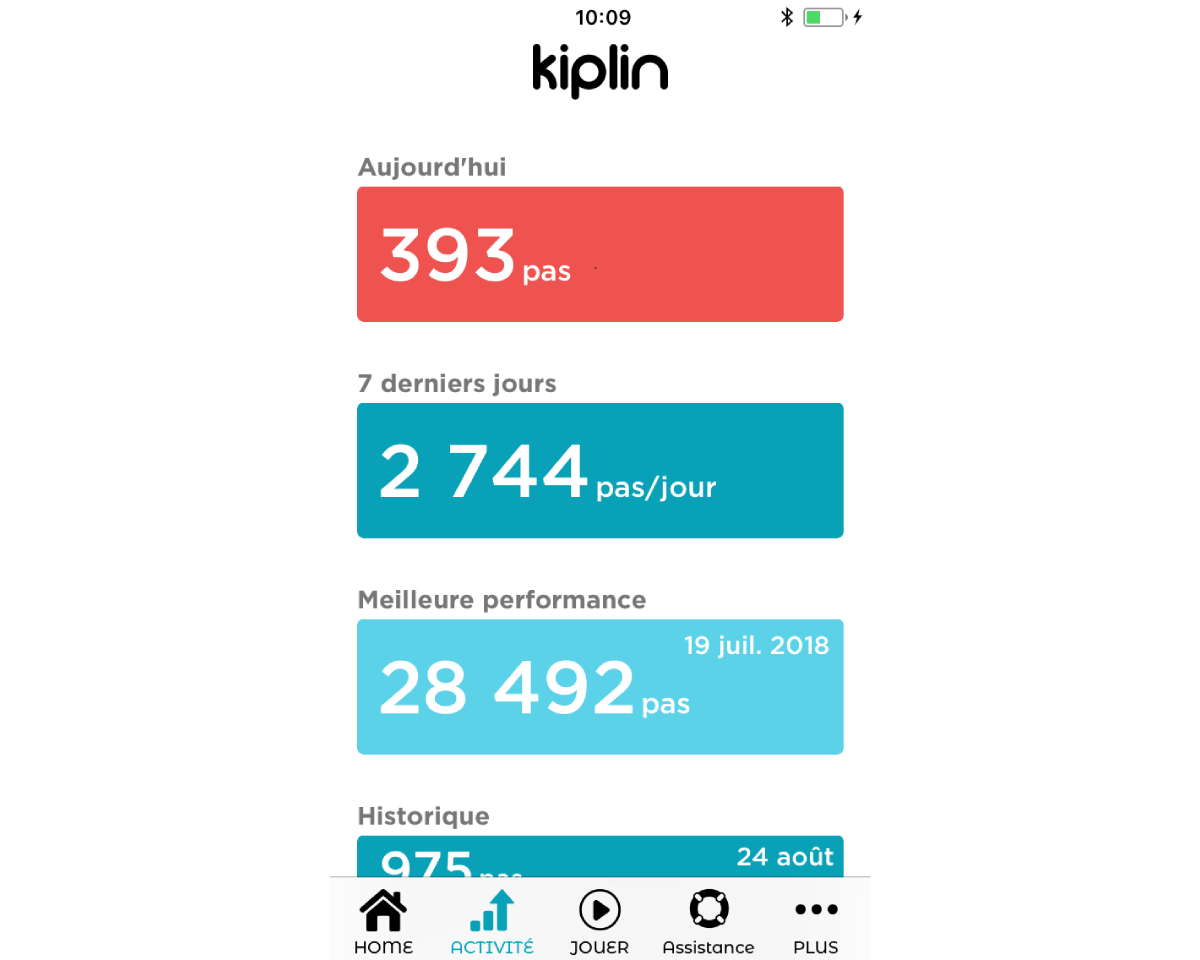
Activity tab (Kiplin’s activity tab example).

The intervention consisted of 2 weeks of the mHealth group challenge. This is a playful challenge developed by Kiplin consisting of (1) a competitive virtual exercise group activity, namely a fictious world tour, (2) participation in a daily chat network with other patients, and (3) access to physical activity information and world tour classification feedback. Patients had a daily goal of doing 6000 steps, recorded by a pedometer. For this challenge, 2 teams were assembled in each group. Details of our adaptation of the mHealth challenge used in a previous study and Kiplin visuals are provided in Multimedia Appendix 4 and Figures 1 and 2.

Informed consent forms were sent by email beforehand to the participants and signed by all participants and the researcher on the day of the first focus group. Participants’ names were not directly linked in any way with the audio recordings. The study received the approval of the national ethics committee (RCB No. 2017-A02062-51).

### Analysis

All focus group sessions were audiorecorded and professionally transcribed verbatim with identifiers removed. In addition, field notes were assembled. We used a grounded theory approach to comprehensively explore and explain the subject, acknowledging that due to our small number of focus groups, back and forth between field work and analyses was limited [39]. Analysis of the focus group data was made using a 3-step process involving (1) reading the transcripts several time to ensure familiarization of the data, reviewing field notes, and creating a codebook based on themes identified and (2) conducting manual thematic content analysis [38] (EM). This was a pilot study with 4 focus groups with 233 minutes total. In this setting, we opted to manually perform thematic content analyses [40]. The research team is highly experienced in manual thematic content analyses. Coding continued until dominant themes that emerged from within the data were clearly identified and the codes from steps 1 and 2 were generalized into broader themes. Data were coded and codes/themes were discussed within the team (EM, IVL [medical oncologist, MD PhD, experienced in survivorship and cancer care], ADM). Our interpretation was submitted to the critical scrutiny of an independent team including psychologists, oncologists, and patient advocates involved in clinical research during a prespecified seminar aimed at presenting the work in progress. After the completion of each focus group, a preliminary analysis was performed to determine the extent to which the information collected was considered sufficiently rich. Descriptive statistics including means, medians, and frequency distributions were used to characterize study participants.

## Results

### Study Participants

Of the 20 patients approached to be enrolled in the study, 9 agreed to participate. Reasons for refusal included unavailability on the predefined date and time for first focus group (n=5), not comfortable using a smartphone (n=3), not a smartphone owner (n=1), distance from research facility (n=1), and pain that prevented exercise practice (n=1). Of the 9 women who participated in the focus groups, 5 were enrolled in the first group of patients (group A) and 4 in the second group of patients (group B). Participant median age at diagnosis was 47 (range 29-60) years, most were married (6/9) and with children (8/9), most lived in towns with more than 20,000 inhabitants (8/9), and all participants were professionally active: 4 clerks, 4 managerial or professional occupations, and 1 with a technician or associate professional position (Table 1).

**Table 1:** Characteristics of the participants.

Characteristics			Focus group session 1 (n=5)		Focus group session 2 (n=4)		Total (n=9)
**Age in years, mean (SD)**			49.6 (7.28)		42.5 (8.07)		46.4 (8.42)
	<40, n	1		1		2	
	40-49, n	2		2		4	
	50-59, n	1		1		2	
	≥60, n	1		0		1	
**Type of town, n**							
	Village (<2000 inhabitants)	0		0		0	
	Town (<20,000 inhabitants)	1		1		2	
	Town (>20,000 inhabitants)	4		3		7	
**Family situation, n**							
	Single	0		1		1	
	Married	3		3		6	
	Divorced	2		0		2	
**Occupational categories, n**							
	Higher professional or manager	2		2		4	
	Manual worker	0		0		0	
	Technician or associate professional	1		0		1	
	Clerk	2		2		4	
	Self-employed	0		0		0	
	Inactive	0		0		0	
**Breast surgery, n**							
	Mastectomy	3		1		4	
	Breast-conserving surgery	2		3		5	
**Lymph node surgery, n**							
	Lymphadenectomy	3		2		5	
	Sentinel node biopsy	2		2		4	
Radiotherapy, n			4		4		8
Chemotherapy, n			5		4		9
Hormotherapy, n			4		4		8
Trastuzumab, n			1		0		1

### Levers and Barriers to Physical Activity and mHealth Use

All patients expressed positive representations of physical activity associating it with physical benefit; nevertheless, some stated feeling that using exercise to reduce fatigue seemed counterintuitive.

The doctor said to me: “Exercise help feeling less fatigued.” So, I told myself: Ok. Well, it’s weird, but I need to walk. Well, I need to do adapted physical activity ... Often, when I woke up in the morning, my knees hurt badly and when we exercise, we can already feel the benefit of it. It hurts less! And this is a little bit counterintuitive because usually, when you feel pain, you decrease your activity.

Rose, 47 years, working part-time

No negative representations of physical activity were conveyed.

A total of 7 overarching themes were identified regarding levers and barriers of physical activity. The 4 main levers identified for physical activity were (1) physical levers including the perception of physical benefit (5/9) and previous practice experience (4/9), (2) psychological levers including the incentive driven by the recommendation of a health care provider (4/9), (3) social levers including the group activity (4/9), and (4) organizational levers with the inclusion of exercise on a regular daily basis (2/9).

The main barriers included were of physical, psychological, and organizational nature. Physical barriers were the late effects of cancer treatment (fatigue; joint and muscle pain; menopausal symptoms; lymphedema; shortness of breath; hand, foot, and mouth syndrome; neuropathy; and weight gain).

It’s hard, and well, I don’t have the right to use my arm since my lymph nodes were removed.

Corinne, 48 years, sick leave

Psychological barriers included lack of motivation, lack of habit, counterintuitive approach, having stopped working out during treatment, fear of being pushed too much, or practicing alone.

Me? Nothing at all. I don’t do sports. I walk but I don’t do physical activity. No incentive to do it.

Sandrine, 44 years, working part-time

Organizational barriers included lack of time, resuming work and/or working full time, and family commitments (Table 2).

I’ve started working again right after treatments, full time. In addition, I have one hour of transportation time.

Marie, 46 years, full-time

Regarding the use of mHealth to be more active, only user-related levers and barriers were identified (eg, psychological levers and barriers). The main lever to the use of mHealth by breast cancer patients was motivation driven by the ability to track activity (3/9), and the main barriers were lack of familiarity, lack of information/explanations, and lack of interest about mHealth (6/9). Table 2 describes themes, messages and quotes from patients regarding levers and barriers of using physical activity and mHealth.

**Table 2:** Levers and barriers to physical activity and mHealth use.

Theme			Message emerging from the analyses	Number of patients citing it	Quotes
**PA^a^ after breast cancer**					
	**Levers**				
		Physical	Physical benefits and previous practice are important levers	Physical benefits 5/9; Previous practice 4/9	And there is another thing that is beneficial too; it’s that I have a lot of joint pain. And indeed, when I move, it hurts less. [Sylvie, 50 years, working part-time] So, it helps the fact that was doing a little bit of physical activity before getting sick. [Rose, 47 years, working part-time]
		Psychological	Oncologist’s recommendation is an incentive	4/9	So, indeed, it’s the oncologist who told me and this made me want to move: “Well, you are tired, there aren’t a hundred options: it’s physical activity!” [Anne, 60 years, sick leave]
		Social	Doing it in a group, with friends, or with relatives are seen as levers	4/9	Because me too, I like doing it in a group. Otherwise it’s hard for me to do physical activity. [Christine, 39 years, working part-time]
		Organizational	Planning PA sessions to fit PA in the daily regular schedule is helpful	Planning PA 2/9	When I come home from work at night ... I feel really exhausted ... So I’m lucky to be able to do physical activity at work at lunch time. [Rose, 47 years, working part-time]
	**Barriers**				
		Physical	Late effects of cancer treatment can negatively impact the practice	Late effects 9/9	And regarding fatigue level, after the end of primary treatments, I was at 10/10. Today, I don’t know, I may be at... it decreased though: I’m at 6/10. But still always with this permanent exhaustion feeling, it is hard to exercise. [Rose, 47 years, working part-time]
		Psychological	Lack of motivation is a main psychological barrier cited	3/9	I’m really not motivated at all. [Sandrine, 44 years, working part-time] So me, I try to do it. But motivation is not always there. [Sylvie, 50 years, working part-time]
		Organizational	Lack of time is a main organizational barrier cited	2/9	But I don’t always have time... It’s also a lack of time. [Rose, 47 years, working part-time]
**Use of mHealth**					
	**Lever**				
		Psychological	To be able to know how many steps a day and track the activity they do is associated with motivation and facilitates the use of these strategies	3/9	It’s a tool that allows us to see what we are doing, either when we don’t do a lot, or when we do a lot. [Sylvie, 50 years, working part-time]
	**Barriers**				
		Psychological	Some patients are not familiar or interested in mHealth, which can be a barrier to their use	6/9	I think I have a friend who has one. But I have never asked for more details. [Marie, 46 years, full-time] I’m not really interested about that. [Marlène, 29 years, sick leave] Oh, me, I’m not a “connected device” person. I’m not a geek at all. [Anne, 60 years, sick leave]

^a^PA: physical activity.

### Kiplin mHealth Group Challenge

All patients felt positively about the Kiplin mHealth group challenge and would recommend such an intervention to other patients and considered it an acceptable proposal. Several positive and negative aspects were identified with the challenge tested. Positive aspects included motivation (7/9), sense of physical and psychological well-being (6/9), promoting good habits (5/9), allowing a group experience (4/9), allowing tracking activity (3/9), and being fun (2/9) (Table 3). Particularly, some patients reported subjective feelings of fatigue improvement.

Personally I think it was a good fatigue ... and I found again, that feeling of sweat pouring from all of my body and that kind of well-being like when I was doing physical activity before [cancer].

Corinne, 48 years, sick leave

It’s not a fatigue that makes you complain, it’s a comforting fatigue.

Marie, 46 years, working full-time

The 4 main negative aspects included lack of information (4/9), challenge is optimized only for walking (4/9), challenge is time-consuming (4/9), and some experienced technical problems (3/9). Challenge modifications implemented by the second group of patients (group B) based on feedback from the first group (group A) included technical simplifications (eg, design changes, improvement of functionalities) and improvement of information tools (eg, FAQ). These modifications resulted in the resolution of some of the negative aspects mentioned by the first group of patients (group A).

**Table 3:** Opinion about Kiplin mHealth group challenge.

Theme and message emerging from the analyses		Number citing it	Quotes
**Preference/advantages**			
	It motivates and push to surpass oneself	7/9	I have to say, it’s really motivating, this thing! It pushes! It pushes you! [Marie, 46 years, working full-time] That suits me perfectly; because it will make me... it will push me! And I am a competitor at heart. [Corinne, 48 years, sick leave] Yes, I think I will take on the challenge. Just by nature! [Sandrine, 44 years, working part-time]
	It makes them feel good (physically and morally)	6/9	I think it was a good fatigue.... And I found again when I was doing physical activity (at the end of practice, when I sweat from every pore), this kind of well-being! [Corinne, 48 years, sick leave] I found benefit regarding the leg pain that I had. And it’s one of the reasons I think that I kept doing it afterward. I’m not saying it’s all gone, but I saw a benefit quite quickly actually. [Sylvie, 50 years, working part-time]
	It generates good habits	5/9	What’s good is that I kept going afterward. So I kept my 6000 steps objective every day. [Sylvie, 50 years, working part-time] I kept the habits afterwards too. And so I keep doing my 6000 steps a day. Well... on average. [Rose, 47 years, working part-time]
	It is a group challenge	4/9	I find it nice, the double objective: in teams and the fact that we move forward together. Because even if we progress in different teams, it’s our cumulative steps that made everyone go forward. [Christine, 39 years, working part-time] Undeniably, I would really recommend working in groups to be physically active again. [Sylvie, 50 years, working part-time]
	It helps quantify their activity	3/9	Me, I found one positive thing, it’s that it objectifies, at least regarding the number of steps we do when we walk. [Anne, 60 years, sick leave]
	It’s fun	2/9	So, everything that’s fun, board games and shared moments, it’s something that drives me. [Corinne, 48 years, sick leave] Me, I like to play, so, I like this! [Sandrine, 44 years, working part-time]
**Obstacles/inconveniences**			
	It’s time consuming	4/9	The main obstacle, it’s the time we can allow to it. [Sylvie, 50 years, working part-time] But me, it still required significant changes on my way of life! Whereas in vacations, it was easy! But at work, personally, I only had on average 800 steps. [Sylvie, 50 years, working part-time] It took a lot of my time! ... The only problem is that I had less time with my children! [Marie, 46 years, working full time] In my opinion, it takes too much time in my life you know. I got back at work not a long time ago. It’s already hard for me since I got back at work to be able to do everything that I need. Because works, it takes a lot of time! And for one and a half years I was on sick-leave. So I feel like I do not have time! [Pascale, 55 years, working part-time]
	Lack of information	4/9	Maybe it would have been useful to explain more. It’s true that we discovered some things when we started talking to each other in the chat box. [Sylvie, 50 years, working part-time] I think that for people like me, who are not used to this kind of thing, it should be explained again, from the beginning, every stage! [Pascale, 55 years, working part-time]
	Only optimized for walking	4/9	So it works inside my bag. It works if I have it in my hand. It works! Except when I go cycling, then it doesn’t work. [Sylvie, 50 years, working part-time] Personally, I was really disappointed that it was not taking my scooter time into account! [Rose, 47 years, working part-time]
	Technical problems	3/9	I was not able to access the app to fill the first mini-game! I had to copy-paste from the internet to open the page. I don’t even remember what I did, but it was complicated. [Marlène, 29 years, sick leave]

## Discussion

### Principal Findings

Physical activity is a well-recognized strategy to improve fatigue after breast cancer, and mHealth can be a good platform to facilitate physical activity. In this study, we focused on a population of inactive breast cancer survivors with documented cancer-related fatigue to, through focus groups, gain in-depth and nuanced insight into participants’ perceptions, opinions, and motivations regarding physical activity and mHealth interventions. After engaging in our mHealth intervention for inactive breast cancer patients with fatigue, several physical activity levers emerged including physical factors (eg, perception of physical benefit and previous practice), psychological factors (eg, motivation increased by provider recommendations), social factors (eg, group practice), and organizational factors (eg, preplanning physical activity sessions). The main barriers to physical activity identified in this study included late effects of cancer treatment, lack of motivation, and lack of time. The lack of familiarity with connected devices was perceived as the main barrier to the use of mHealth as a mean to promote physical activity. The tested mHealth group challenge was associated with several positive representations including well-being, good habit promotion, and motivational catalyzer.

First, the barriers to physical activity practice that were identified mostly aligned to what has been previous presented in the literature. The main barriers for breast cancer survivors to engage in physical activity reported in the literature include organizational barriers, with a substantial proportion of patients reporting lack of time or lack of access to facilities, physical factors including late and long-term effects of cancer treatment, and social/psychosocial factors such as lack of motivation or lack of social support [20,41-43].

Second, as previously shown in literature, peer support in a group was seen as an important incentive to physical activity practice, having a positive impact on both initiation and persistence of these kinds of behavioral changes [42,44]. In our population, one of the main levers of engagement in and pursuit of physical activity was perceiving physical benefit (eg, reduction of joint pain was considered an incentive to maintain physical activity). In addition, perceived benefits in weight and health management, improvement of body image, personal fulfillment, regaining normality, positive beliefs about efficacy and outcomes, and positive emotions (eg, enjoyment) also seemed to play roles as levers [20,42,45].

Third, although several mHealth interventions for breast cancer patients targeting physical activity or cancer-related fatigue have been conducted [29-31,36,46-51], to our knowledge, none of them has examined levers and barriers to physical activity among cancer patients with cancer-related fatigue as a primary outcome within the context of an mHealth intervention. In previous studies in the overall population, there were 3 main barriers for patients to engage with mHealth: user-related barriers (eg, lack of digital literacy, lack of motivation), health-related barriers (eg, late effect of treatments, lack of physical ability), and technology-related barriers (eg, technical problems, intrusiveness) [52,53]. In our population, we found similar obstacles. In addition, the literature also presents several levers/facilitators to engage with mHealth among cancer patients that were also identified in our population: user-related levers (eg, planning physical activity, motivation, self-efficacy, social support), health-related levers (eg, feeling good), and technology-related levers (eg, convenience, tailoring of the intervention, ease of use) [52,53]. Some solutions to reduce barriers to physical activity and to the use of mHealth are presented in Table 4.

These findings suggest that mHealth can be an acceptable option to promote physical activity in this population of breast cancer survivors. mHealth is emerging as way to monitor patient-reported outcomes and promote health behavior improvement for a large proportion of patients. Wearable devices (eg, phone or pedometer) are an effective strategy to increase physical activity [54]. With phones having a growing importance in our lives, app-based mHealth interventions can be a good way to help patients. mHealth offers a new way to propose cost-effective health care interventions; indeed, app-based or web-based interventions allow care to be accessible to an increasing number of people outside of the hospital [49]. Several mHealth apps for cancer patients have been developed these past few years, and some are being tested in clinical trials [52-55]. Acceptability of mobile phone apps has been shown to be high among users [53]. Participant engagement with the challenge was substantial; nevertheless, our challenge was short and prior literature suggests a decrease in adherence to these solutions over time [56-58]. Therefore, when using these strategies to help exercise engagement among breast cancer patients with fatigue, it will be important to include elements such as the usability of the technology, motivating factors, data monitoring, personal contact with the study personnel/support, and personalized feedback that has shown before to contribute to better adherence [59].

Even if mHealth solutions are used by a large number of people and are a good tool to use for some populations, we acknowledge that not all types of patients are interested in or able to use them. Thus, alternative nonvirtual offerings may also be required. Regarding physical activity and fatigue after breast cancer, joining an association offering adapted physical activity for cancer patients, engaging with a personal trainer, practicing in a group or with a family member, participating in group counseling, or using self-monitoring and goal setting may be effective solutions.

**Table 4:** Solutions to reduce barriers.

Barrier			Solution
**Barriers to physical activity**			
	Joint pain and fatigue	Explain that being physically active can help reduce joint pain and fatigue	
	Reduced motivation	Offering rewards inside the challenge for regularity and improvements	
	Lack of time (eg, working again, family commitment)	Show different ways to gain steps each day without needing a lot of time (eg, leaving the bus/metro one stop early, parking the car farther away from the workplace/stores, taking the stairs, using the bathroom on another floor at work). Help participants to find ways of freeing some time	
	Counterintuitiveness of being active when fatigued or feeling pain	Explain that being physically active can help reduce joint pain and fatigue; tell them they will likely feel it after a few days	
**Barriers to the use of mHealth and challenge improvements**			
	Complexity of the device’s use	A simpler way to record steps	
	Device only adapted to walking	An only device recording all kind of physical activity (cycling, swimming, etc)	
	Visibility of new messages in the chat	Build a pop-up alert when a new message is posted in the chat	
	Visibility of the itinerary (world tour) of the challenge on a mobile device	Seeing the map of the challenge more clearly on the phone (world tour) or finding another way to present it	
	Information about step counts	A day-by-day recap of the step count	
	Reduced motivation	Add more mini-games, more interactions between participants	
	Playing with strangers	Use the challenge in groups that already know each other	

The first version of the Kiplin mHealth group challenge was web-based and used a pedometer to record step count; they then developed an app-based challenge with a built-in step-counter. Kiplin adapted the mHealth challenge to this population of breast cancer patients with fatigue by decreasing the number of steps to reach per day. The tailoring of the intervention to several kinds of populations may ensure feasibility and adherence. Indeed, patients were satisfied after participation in the challenge and gave positive feedback. The group challenge that we exposed our patients to was seen as motivational, fun, and a good way to track steps; in addition, it generated good habits and made women feel good both physically and emotionally. Our study suggests that this kind of challenge might be a good way to engage patients to be physically active after the end of treatment, with the group-based objectives and games acting as ways to make physical activity less difficult, more attractive, and motivational for patients. Kiplin’s mHealth group challenge may be a way to overcome some of the barriers to engaging in physical activity commonly encountered such as access, motivation, and social support. It can also help overcome some of the barriers to engaging in mHealth technology; some troubleshooting and technical support was provided along the course of the challenge to patients who were experiencing difficulties with app settings or overall functioning.

### Limitations

We acknowledge our study has limitations. First, this was an exploratory study with limited sample size, so even if we discovered a range of barriers and levers represented in our focus groups and found some redundancy, the generalizability of study findings might be limited, and these preliminary data should be further investigated in a randomized controlled trial. Second, participants were predominantly college-educated, and this may constitute another limit to the generalizability of our results. Third, we acknowledge selection bias performed both by providers (they may have been inclined to pick well-disposed patients) but also regarding patient acceptability (those not being comfortable using a smartphone or not owning a smartphone are likely to have refused participation in the study). Fourth, the duration of the challenge was limited to a 2-week period, and conducting a study for a longer period may lead to collecting different perceptions from patients. Thus, making assumptions of efficacy of the intervention in question is not possible. Finally, we tested a specific intervention, and perceptions can be different if a different mHealth intervention is used.

### Conclusion

Kiplin’s mHealth group challenges were perceived as levers for the practice of physical activity in this population. This qualitative exploration aided the improvement of the challenge. mHealth group challenges should be explored as options to promote physical activity in a population with fatigue after breast cancer.

## Appendix

Multimedia Appendix 1 Focus group session 1 script.

Multimedia Appendix 2 Focus group session 2 script.

Multimedia Appendix 3 Sociodemographic questionnaire.

Multimedia Appendix 4 Kiplin solution development, adapted.

## Abbreviations

COREQ: Consolidated Criteria for Reporting Qualitative Studies
mHealth: mobile health
QoL: quality of life
