# Supplementary material for: Use of mHealth to Increase Physical Activity Among Breast Cancer Survivors With Fatigue: Qualitative Exploration
Source: JMIR Cancer. 2021 Mar 22;7(1):e23927. doi: 10.2196/23927 (PMC8088868; doi:10.2196/23927)
Supplement: Multimedia Appendix 1 [file cancer_v7i1e23927_app1.docx]

**Multimedia Appendix 1**

**Focus group script (1st Focus group)**

**Opening**

Welcome participants. Thank participants. Present the team and the objectives of the study. Remind of the confidentiality of the discussions and the anonymity. Present myself and the moderators present in the room. Present the development of the focus group. Ask if they have questions.

1. **First part of the Focus Group**

**First topic**

Today, we are increasingly hearing speak about benefits of doing regular physical activity for our health. We all know not everybody has these kinds of habits. How do you lie regarding this?

*If needed as follow-up questions:*

*Do you practice a physical activity? Which one? How much time/how many times a week?*

*What are the barriers to your physical activity practice?*

**Second topic**

You may have heard about mobile or internet app and software but also about “connected devices” developing in the health field. What are you thinking about this as a support to physical activity practice?

*If needed as a follow-up question:*

*Is it a way to help worth promoting?*

1. **Second part of the focus group**

**Presentation of the mHealth challenge** by a member of Kiplin®.

**Setup mobile phone** with the Kiplin® app, link it to the build in steps count.

**Closing**

Ask if they have comments or questions.

Remind researcher’s contact details.

Review the next steps (2-week challenge, objective (6000 steps), and date of the next focus group).

Thank the participants.
