# Supplementary material for: Use of mHealth to Increase Physical Activity Among Breast Cancer Survivors With Fatigue: Qualitative Exploration
Source: JMIR Cancer. 2021 Mar 22;7(1):e23927. doi: 10.2196/23927 (PMC8088868; doi:10.2196/23927)
Supplement: Multimedia Appendix 2 [file cancer_v7i1e23927_app2.docx]

**Multimedia Appendix 2**

**Focus group script (2^nd^ Focus group)**

**Opening**

Welcome participants. Thank participants. Present the development of the focus group. Remind of the confidentiality of the discussions and the anonymity. Ask if they have questions.

1. **First part of the Focus Group**

**First topic**

- You have participated to the “mHealth connected group challenge” developed by Kiplin®, can you briefly describe how things went for you during the 2-week challenge?

**Second topic**

- What are you impressions regarding the program you participated in?

*If needed as follow-up questions:*

- *What were the advantages of this experience, the things you liked the most?*
- *What were the things you liked less? Were there things that got in the way of you use?*
- *Beside the challenge itself, were there things in your daily life that have been an obstacle to your participation?*
- *What are the elements that can be improved? Do you have suggestions to do so?*
- *What was you impression before doing the challenge, a priori, just after doing the 1^st^ focus group? Has it evolved?*
- *What did you think about the “connected device”? About the* “*group” aspect? About the “game” aspect?*

**Third topic**

- What did this challenge brought to you in terms of benefits/changes?

*If needed as follow-up questions:*

- *What was the impact of this challenge on your level of physical activity?*
- *What was the impact of this challenge on your fatigue?*
- Would you recommend this program to other patients to help them get physically active?
- What motivated you to participate to the study?

**Closing**

Ask if they have comments or questions.

Thank the participants.

Briefly resume the next steps of the study.
