# Supplementary material for: Use of mHealth to Increase Physical Activity Among Breast Cancer Survivors With Fatigue: Qualitative Exploration
Source: JMIR Cancer. 2021 Mar 22;7(1):e23927. doi: 10.2196/23927 (PMC8088868; doi:10.2196/23927)
Supplement: Multimedia Appendix 3 [file cancer_v7i1e23927_app3.docx]

**Multimedia Appendix 3**

**Socio-demographic and clinical questionnaire**


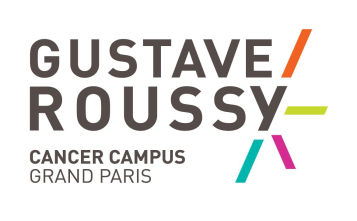
**Feasibility study of the use of mHealth to promote physical activity and reduce fatigue after breast cancer treatment**

ETUDE FATI-mHEALTH; CSET N°2017-2616; N°ID-RCB: 2017-A02062-51

Promoter: Gustave Roussy

Principal Investigator: Dr Ines VAZ-LUIS

**Socio-demographic and clinical questionnaire**

**Alias in the challenge:** ………………………………………………………

- How old are you?
- What is your educational background?
- What is your profession (be precise)?
- Are you currently working?
- What is your family situation (e.g. married, single, divorced, widowed)?
- Do you have children (how many)?
- Where are you living (big city, medium-sized city, small town, village)?
- Did you had breast and/or lymph node surgery, did you receive chemotherapy, did you receive radiotherapy, did you receive Trastuzumab, are you taking hormonotherapy?
- When did your last treatment/intervention ended (hormonotherapy excluded)?
