# Supplementary material for: Use of mHealth to Increase Physical Activity Among Breast Cancer Survivors With Fatigue: Qualitative Exploration
Source: JMIR Cancer. 2021 Mar 22;7(1):e23927. doi: 10.2196/23927 (PMC8088868; doi:10.2196/23927)
Supplement: Multimedia Appendix 4 [file cancer_v7i1e23927_app4.docx]

**Multimedia Appendix 4**

**Kiplin® solution development**

Kiplin®'s initial project was to promote well-being and quality of life at work through playful and collective connected physical activity programs aimed at coworkers. The challenge choice was done after internal reflections and technological watch about what kind of following was possible regarding physical activity. Step number was chosen as physical activity unit of measurement due to its simplicity (it’s understandable by everyone) and its accessibility from most connected devices (contrary to other metrics such as the number of minutes of physical activity). The choice of a virtual journey was selected due to its narrative capacity that allowed to easily tell a motivating and inspiring collective story. Several virtual journeys were proposed such as a world tour, crossing the United States, from the Earth to the Moon, the Paris-Brest-Paris (a French cycling competition), the Vendée Globe (a French sailing competition), and even a virtual journey customizable by the client.

Kiplin®'s solution is focused on delivering digital group challenges that provide participants the opportunity of engaging in exercise. These challenges aim to stimulate group exercise, by having common competitive goals. Quiz, chatting to share tricks or tips are incorporated. This solution is on the market since 2016 and in the beginning, it was used with two focus: 1) as a team-building tool which was financed by employers and addressed to employees and 2) as a loss of autonomy prevention tool addressed to the elderly at risk.

In 2017, Kiplin® was approached by health facilities and health professionals, facing difficulties in engaging their patients in the practice of physical activity, who saw in the solution an interesting approach to promote daily physical activity among their patients. Thus Kiplin®’s solution started to be adapted as an intervention to be used by cancer patients to promote exercise practice. In this domain, it was first pretested in two distinct settings:

1. the first involving nine breast cancer patients from a French private hospital after the end of their primary treatment that took place from April to July 2017. Patients were invited by their physiotherapist to walk 10.000 steps a day, were equipped with a connected device to record their steps number, and were put in contact with each other using Kiplin®’s chatbox. No objective measure of efficacy, but a qualitative evaluation suggested acceptability of the solution and subjective perception that it helped to promote exercise.
2. the second involving a public French Hospital with 50 patients with different cancer types and at different moments at their disease (during and after treatment) and 50 providers that took place in October 2017. Patients and staff were mixed into teams of 5 and faced challenges based on their daily physical activity. The challenge lasted 4 weeks. The aim was to observe physical activity evolution (step number), patient’s satisfaction (questionnaire filled at the end of the study), QoL (evaluated by the MOS 36-item short-form health survey before and after the intervention). Qualitative results were very encouraging, the experience was perceived as positive favoring both physical activity and social interactions and improved patient's self-efficacy. All the participants who completed the final satisfaction questionnaire stated that they would recommend such an intervention. Objectively, the daily number of steps increased as compared to baseline. A perceived impact on both "vitality" and "social life and relationship with others" was also observed, as assessed by the MOS 36-item short-form health survey*.* Suggestions to the solution were integrated.

The positive preliminary results of those experiments, in terms of patients acceptability and physical activity level, confirmed the potential of the solution within a therapeutic field. These experiences led to a version of the Kiplin® solution with a focus on cancer patients. Since then, Kiplin® has integrated healthcare skills and the priority strategy is to develop the solution as a digital therapy for patients with chronic disease (mainly cancer, obesity and diabetes).

**Kiplin® solution’s adaptation to our study**

The intervention used in our study was based on this version of the solution but it was adapted through a collaboration between: 1) the multidisciplinary team of Gustave Roussy involving researchers, medical oncologists, sociologists and psychologists 2) Kiplin® team involving exercise physiologists and engineers and 3) patients advocates. The following parameters were reviewed and defined in this multidisciplinary effort:

- Narrative story: The team opted to design a world tour challenge considering that this pick would be attractive and stimulating to patients, facilitating their retention in the challenge.
- Quizzes, chats and functionalities.
- Daily step goal for patients: Although the WHO recommendation for daily activity is 10 000 steps, given the focus on a breast cancer population with moderate to severe fatigue and upfront sedentary behavior we decided to establish a 6000 steps daily focus.
- Challenge duration: given that our main goal was to explore representations, levers and barriers to physical activity and mHealth interventions and an exploratory evaluation of the satisfaction of the Kiplin® solution we deemed that a two week challenge would be sufficient.

This intervention was pre-tested by some team members, including our patient advocates, before beginning of the study.
